# A standard photomap of the ovarian nurse cell chromosomes for the dominant malaria vector in Europe and Middle East *Anopheles sacharovi*

**DOI:** 10.1186/s12936-018-2428-9

**Published:** 2018-07-30

**Authors:** Gleb N. Artemov, Alena I. Velichevskaya, Semen M. Bondarenko, Gayane H. Karagyan, Sargis A. Aghayan, Marine S. Arakelyan, Vladimir N. Stegniy, Igor V. Sharakhov, Maria V. Sharakhova

**Affiliations:** 1 Laboratory of Ecology, Genetics and Environment Protection, Tomsk State University, Tomsk, Russia; 2 Scientific Center of Zoology and Hydroecology, The National Academy of Sciences of the Republic of Armenia, Yerevan, Armenia; 3 Chair of Zoology, Yerevan State University, Yerevan, Armenia; 4 Department of Entomology, Fralin Life Science Institute, Virginia Tech, Blacksburg, VA USA

**Keywords:** *Anopheles sacharovi*, Mosquito, Gytogenetic map, Fluorescence in situ hybridization

## Abstract

**Background:**

*Anopheles sacharovi* is a dominant malaria vector species in South Europe and the Middle East which has a highly plastic behaviour at both adult and larval stages. Such plasticity has prevented this species from eradication by several anti-vector campaigns. The development of new genome-based strategies for vector control will benefit from genome sequencing and physical chromosome mapping of this mosquito. Although a cytogenetic photomap for chromosomes from salivary glands of *An. sacharovi* has been developed, no cytogenetic map suitable for physical genome mapping is available.

**Methods:**

Mosquitoes for this study were collected at adult stage in animal shelters in Armenia. Polytene chromosome preparations were prepared from ovarian nurse cells. Fluorescent in situ hybridization (FISH) was performed using PCR amplified probes.

**Results:**

This study constructed a high-quality standard photomap for polytene chromosomes from ovarian nurse cells of *An. sacharovi*. Following the previous nomenclature, chromosomes were sub-divided into 39 numbered and 119 lettered sub-divisions. Chromosomal landmarks for the chromosome recognition were described. Using FISH, 4 PCR-amplified genic probes were mapped to the chromosomes. The positions of the probes demonstrated gene order reshuffling between *An. sacharovi* and *Anopheles atroparvus* which has not been seen cytologically. In addition, this study described specific chromosomal landmarks that can be used for the cytotaxonomic diagnostics of *An. sacharovi* based on the banding pattern of its polytene chromosomes.

**Conclusions:**

This study constructed a high-quality standard photomap for ovarian nurse cell chromosomes of *An. sacharovi* and validated its utility for physical genome mapping. Based on the map, cytotaxonomic features for identification of *An. sacharovi* have been described. The cytogenetic map constructed in this study will assist in creating a chromosome-based genome assembly for this mosquito and in developing cytotaxonomic tools for identification of other species from the Maculipennis group.

## Background

*Anopheles sacharovi,* Favr [1] is one of the Palearctic members of the Maculipennis group of malaria mosquitoes that also includes *Anopheles atroparvus*, *Anopheles artemievi, Anopheles beklemishevi*, *Anopheles daciae*, *Anopheles labranchiae*, *Anopheles maculipennis*, *Anopheles martinus*, *Anopheles melanoon*, *Anopheles messeae*, and *Anopheles persiensis* [2]. Four of these 11 species, *An. atroparvus*, *An. labranchiae*, *An. messeae*, and *An. sacharovi,* are considered dominant malaria vectors in Eurasia [3]. Among them, *An. sacharovi* has the most southern distribution and is the most dangerous malaria vector that transmits malaria in South Europe and in the Middle East [3]. Currently, *An. sacharovi* is involved in transmission of vivax malaria in Iran [4–6], Iraq [7] and Turkey [8]. After the collapse of the Soviet Union, this species became responsible for malaria re-emergence in Georgia [9], Armenia [10, 11] and Azerbaijan [12]. The success of *An. sacharovi* as a malaria vector is a result of a highly plastic adaptation of this species at both adult and larval stages [3]. It can breed in different water reservoirs, such as swamps, marshes, river margins, streams, pools, and ditches. Female mosquitoes have opportunistic blood-feeding behaviour and can feed on any available host including human, cow, sheep, chicken, horse, and donkey. It has been demonstrated that *An. sacharovi* is resistant to DDT [13] and dieldrin [5]. Such ecological and behavioural plasticity, together with the emerged insecticide resistance, prevented elimination of *An. sacharovi* during several anti-vector campaigns conducted in Israel, Greece and Turkey. Additionally, global warming raises concerns about the possible spread of *An. sacharovi* to the dry areas and territories with high altitudes [14].

An importance of *An. sacharovi* as a malaria vector stimulates studies of this species from different perspectives including cytogenetic analyses. The first cytogenetic map for this species was developed based on photo images of lacto-aceto-orcein-stained polytene chromosomes from salivary glands [15]. This map was used for species identification of *An. sacharovi* and for the cytotaxonomic validation of the species status of *An. martinius* Shingarev originally described in Uzbekistan in 1926 [16]. *Anopheles martinius* is identical to *An. sacharovi* based on external characters including egg morphology, and its name for a long period of time was considered a synonym for *An. sacharovi* [17]. The cytogenetic analysis clearly demonstrated that *An. sacharovi* and *An. martinius* differ from each other by two fixed paracentric inversions on chromosomes X and 3L and, thus, represent two different species [15]. Moreover, polytene chromosomes of laboratory hybrids between *An. sacharovi* and *An. martinius* demonstrated partial asynapsis of the homeologous chromosomes, which is typical for the interspecies hybrids in other species of Diptera [18]. The importance of this finding was emphasized by G. B. White in his review of systematic reappraisal of the *An. maculipennis* complex [19]. Later, the species validity of both *An. sacharovi* and *An. martinius* was supported by fixed nucleotide substitutions in the internal transcribed spacer 2 (ITS2) [20]. Chromosome polymorphism has not been yet detected in natural populations of *An. sacharovi*.

In addition to species diagnostics, the cytogenetic map of *An. sacharovi* was utilized to study chromosome evolution and phylogeny of Palearctic members of the Maculipennis group [21]. The analysis of overlapping chromosomal inversions in 7 sibling species from the *An. maculipennis*  complex revealed three branches of the phylogenetic tree: *An. atroparvus*-*An. labranchiae*; *An. melanoon*-*An. maculipennis*-*An messeae*; and *An. sacharovi*-*An martinius.* Among them, the branch *An. atroparvus*-*An. labranchiae* was considered as the basal. Although the phylogeny based on ITS2 sequences supported the presence of 3 major clades in the Palearctic group, the molecular analysis suggested that *An. sacharovi* is the basal lineage [20, 22, 23]. Thus, the phylogeny of the Maculipennis group remains unresolved.

The genomics era offers new opportunities for the development of modern genome-based strategies for vector control [24, 25] including the CRISPR/Cas9-based gene-drive technologies [26]. Following the major malaria vector in Africa *Anopheles gambiae* [27], the genomes for other malaria mosquitoes have been sequenced [28–30]. The availability of cytogenetic maps allows the development of high-quality genome assemblies anchored to the chromosomes. However, only 5 chromosome-based genome assemblies have been developed for malaria mosquitoes [28, 29, 31, 32], including one for the dominant vector of malaria in Europe *An. atroparvus* [33]. The comparison of the chromosome-based assemblies provided important insights into chromosomal evolution in the genus *Anopheles*. A high rate of the sex chromosome evolution [34], whole-arm translocations among autosomes [35], and inter-arm rearrangements [32] have been reported.

In this study, a standard cytogenetic photomap for *An. sacharovi* was developed. The suitability of this map for physical mapping was demonstrated by placing five PCR-amplified genes to the chromosomes of this species. In addition, cytogenetic landmarks that can be used for cytotaxonomic identification of *An. sacharovi* were described. This new map will assist in the development of the chromosome-based genome assembly for this important malaria vector.

## Methods

### Mosquito collection

About 250 adult specimens of *An. sacharovi* were collected in September 2015 in the horse and pig shelters in Araksavan village, Ararat region, Armenia (39°59′58.5″N, 44°27′38.9″E). Ovaries from half-gravid females were dissected, placed in individual 0.5-ml tubes with cold Carnoy's fixative solution (ethanol: acetic acid in 3:1 ratio), and stored at − 20 °C.

### Chromosome preparation

Ovarian nurse cells polytene chromosome preparation was done following a standard procedure [33, 36]. A piece of ovary was placed on the slide into a drop of 50% propionic acid for 10 min, covered by the coverslip and squashed. Chromosome preparations with a high-quality banding pattern were selected for the image acquisition and fluorescence in situ hybridization (FISH). High-quality preparations were dipped in liquid nitrogen, the coverslips were removed, and the preparations were dehydrated in an ethanol series (50, 70 and 100%), air dried and stored at room temperature.

### Chromosome map development

Images for the chromosome map were obtained using a phase contrast AxioImager A1 microscope (Carl Zeiss, OPTEC LLC, Novosibirsk, Russia), CCD camera MrC5, and AxioVision 4.8.1 software (Carl Zeiss, OPTEC LLC, Novosibirsk, Russia) at the 100x magnification. The images were combined, straightened, shaped, and cropped using Adobe Photoshop CS2 [33, 36]. The marking of nurse cell chromosomes by divisions and sub-divisions was conducted using a previously developed salivary glands chromosome map [15].

### PCR of DNA probes

Mosquito DNA was extracted from specimens using standard protocol for Qiagen DNeasy Mini Kit (Qiagen, Germantown, MD, USA). Gene-specific primers were designed to amplify four unique exon sequences using PRIMER-BLAST software available at the NCBI website [37]. Genes from different chromosome arms of *An. sacharovi* were selected based on similarity with the *An. atroparvus* genome [28, 33, 36]. The primer design was based on gene annotations from the AatrE1 genome assembly available at the VectorBase website [38]. PCR was performed in the presence of 1xPCR buffer (SibEnzyme Ltd., Novosibirsk, Russia), 2.5 mM magnesium chloride (SibEnzyme Ltd., Novosibirsk, Russia), 0.2 mM dNTP (Thermo Fisher Scientific TM, USA), and 0.02 u/μl Taq Polymerase (SibEnzyme Ltd., Novosibirsk, Russia).

### DNA-probe labelling and fluorescence in situ hybridization (FISH)

Gene-specific DNA-probes were labelled using a Random Primer Labeling Protocol: 25 μl of labelling reaction contained 50-ng DNA, 1x Klenow buffer (Thermo Fisher Scientific TM, USA), 44-ng/μl Exo-Resistant Random Primer (Thermo Fisher Scientific TM, USA) 0.1-mM dATP, dGTP, and dCTP and 0.015-mM dTTP, 0.016-mM TAMRA-5-dUTP, and 5 units of Klenow fragment (Thermo Fisher Scientific TM, USA) in a PCR tube. The required amounts of DNA, Klenow buffer, and Random Primers were mixed, brought up to 12 μl with water, and heated at 95 °C for 5 min in a thermocycler. The solution was chilled on ice, and appropriate amounts of nucleotides, Klenow fragment and water were added to reach 25 μl. The reaction mix was incubated at 37 °C for 18 h. FISH was performed using previously described standard protocol [39, 40].

## Results

### The development of a standard cytogenetic map for *Anopheles sacharovi*

**Fig. 1:**
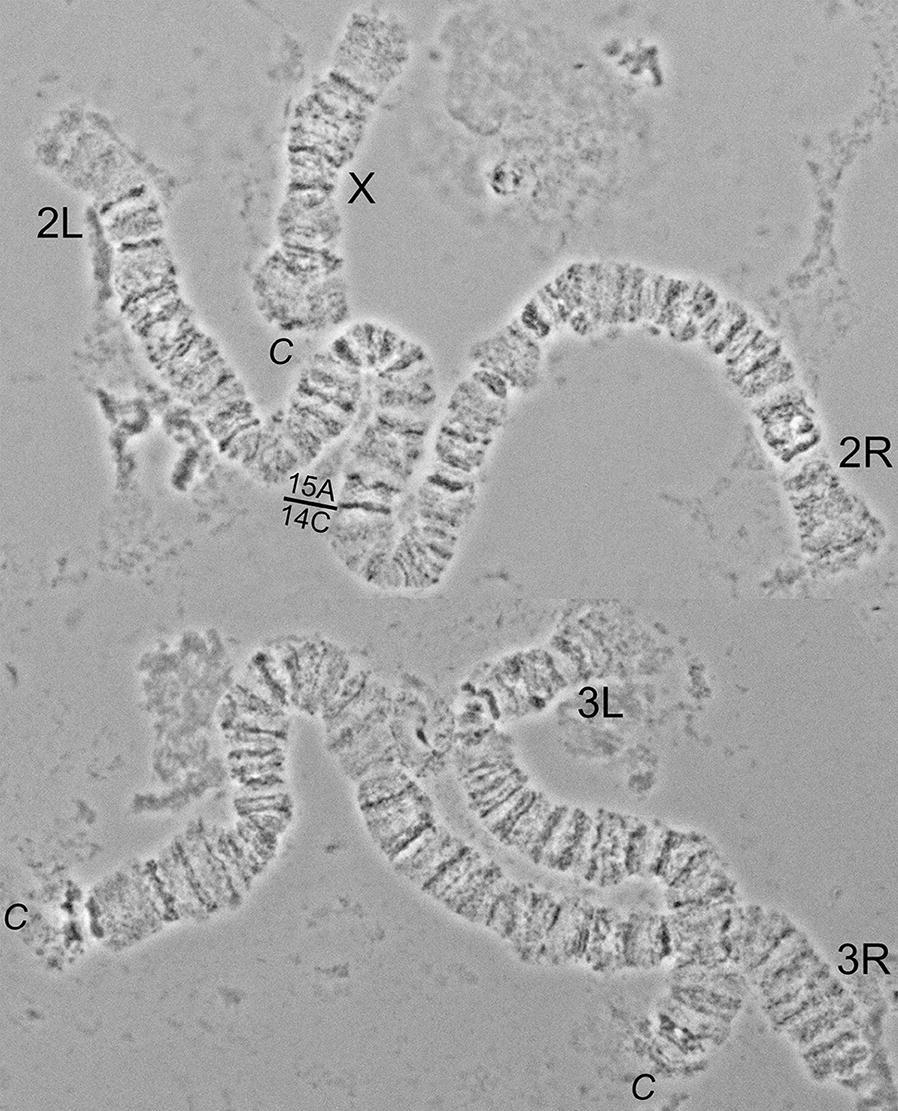
A complement of *Anopheles sacharovi* nurse cells polytene chromosomes. The X chromosome is marked as X. 2R, 2L, 3R, and 3L stand for autosome arms. The boundary between the right and the left arm of chromosome 2 indicated by a solid line. Centromeric regions of X chromosomes, 3R and 3L arms are indicated as *C*

In this study, a high-quality photomap was developed for the polytene chromosome from the ovarian nurse cells of a dominant malaria vector in Europe and Middle East, *An. sacharovi*. As was shown before [15], chromosome complement of *An. sacharovi* consists of 3 pairs of chromosomes in diploid cells that correspond to 5 polytene chromosome arms in ovarian nurse cells (Fig. 1). The X chromosome is represented by a single arm. Arms of chromosome 2 are usually connected together, whereas arms of chromosome 3 get separated from each other during squashing of chromosomal preparations because of fragile connections between them [41, 42]. Relative lengths of the chromosomes in *An. sacharovi* (Table 1) are similar to that in *An. atroparvus* [33]. The X chromosome is the shortest arm, and the 3R arm is the longest arm in the polytene chromosome complement. The banding pattern and quality of *An. sacharovi* chromosomes are similar to that in *An. atroparvus* [33].

**Table 1:** Measurements and proportions of the polytene chromosomes in the ovarian nurse cell of *Anopheles sacharovi*

Chromosomes	X	2	3
Average length, μm	136.6	757.53	815.0
Relative chromosome length, %	8.1	44.4	47.9
Relative arm length, %	n/a	48.6	37.1

**Fig. 2:**
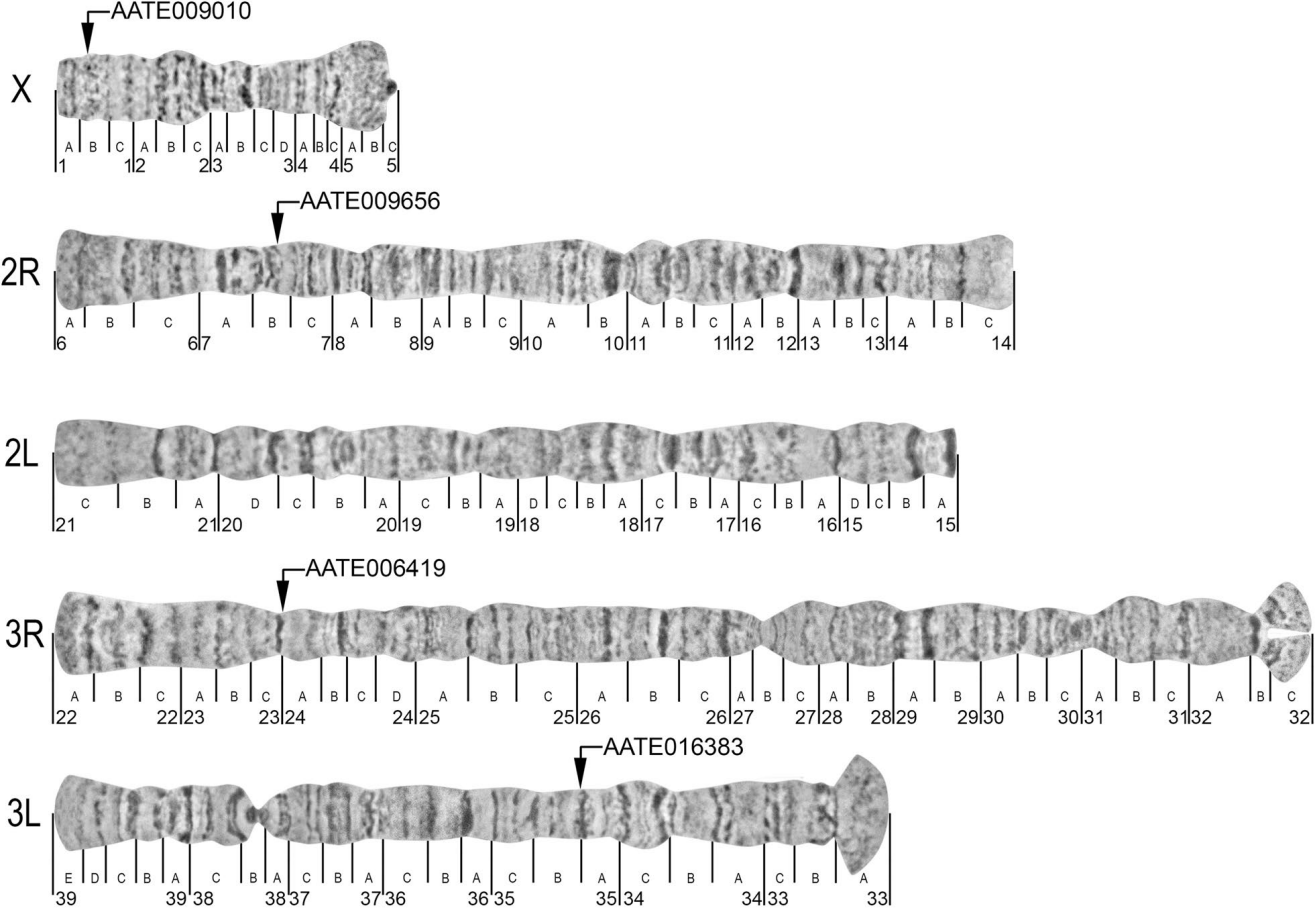
A standard cytogenetic photomap for the polytene chromosomes from ovarian nurse cells of *Anopheles sacharovi*. Chromosome arms are indicated as X, 2R, 2L, 3R, and 3L at the telomere ends of the chromosomes. The lines below the chromosomes indicate the boundaries of numbered and lettered divisions and sub-divisions, respectively. The positions of four genes AATE009010, AATE009656, AATE006419, and AATE016383 on the cytogenetic map are indicated by arrows

To develop a cytogenetic map for *An. sacharovi*, ~ 150 chromosome preparations from ovarian nurse cells of 76 specimens were screened for the presence of high-quality polytene chromosomes with clear banding structure. Ten to 15 images of each chromosome arm were selected and further processed for map development. Chromosome images were combined, straightened, shaped, and cropped using Adobe Photoshop CS2 [33, 36]. Following the nomenclature previously developed for the salivary gland chromosomes of *An. sacharovi* [15], ovarian nurse chromosomes were sub-divided into 39 numeric divisions and 119 lettered sub-divisions (Fig. 2). The order of lettered sub-divisions for the 2L and 3L arms in the new map was changed because in the previous map the order of lettered sub-divisions was opposite to the order of numbered divisions. This discrepancy has been corrected, and both numbered and lettered divisions now follow the same order for all 5 chromosome arms in the new map.

Chromosome arms of *An. sacharovi* can be recognized easily by the following landmarks or regions with specific banding patterns. The X chromosome is the shortest in the chromosome complement. It is distinguishable by a diffused granulated structure of the region 4C-5B that is terminated by a dot-like compact dark band in region 5C. No variability was found in condensation of the diffuse chromatin or in morphology of the terminal dark band. However, variability in the pericentromeric region is typical for *An. atroparvus* X-chromosome [33].

Chromosome arm 2R can be recognized by the flared telomere end with a distinct banding pattern. The pericentromeric region in 2R forms a puffy area in region 14C that has a diffuse structure with no bands. A set of three dark narrow bands in region 10A and 2 dark bands in region 10B can be considered an additional landmark for the middle of 2R arm chromosome. The telomere region of the 2L arm has a long light area limited by 2 thin bands in region 21C and a dark band in region 21B. In contrast to the light-diffused pericentromeric region in 2R arm, 2L arm contains two dark bands in region 15A–15B.

In contrast to the telomere ends in chromosome 2, the telomere end in chromosome arm 3R is striped, and it starts with a dark band in region 22A followed by 6 additional distinct bands in region 22A–23B. Pericentromeric region 32B–32C of 3R arm is asynaptic in ~ 60% nuclei, but homologous chromosomes are always paired in region 32B that contains a dark band. Three dark bands in regions 26A, 26B and 26C that localize at an equal distance from each other and are followed by a thin, light region in 27BC and can also be used for recognizing the 3R arm. Chromosome arm 3L is the shortest autosomal arm that, in contrast to 3R arm, has a flared light telomere end. The pericentromeric region usually forms a fan consisting of diffuse chromatin fibrils. In addition, 3L contains a typical landmark for all members of the Maculipennis group in region 38A–38B, known as ‘bird eye’, represented by a set consisting of a dark crescent-shaped band followed by a dot-like band.

### Physical mapping of orthologous genes to *Anopheles sacharovi* chromosomes

**Fig. 3:**
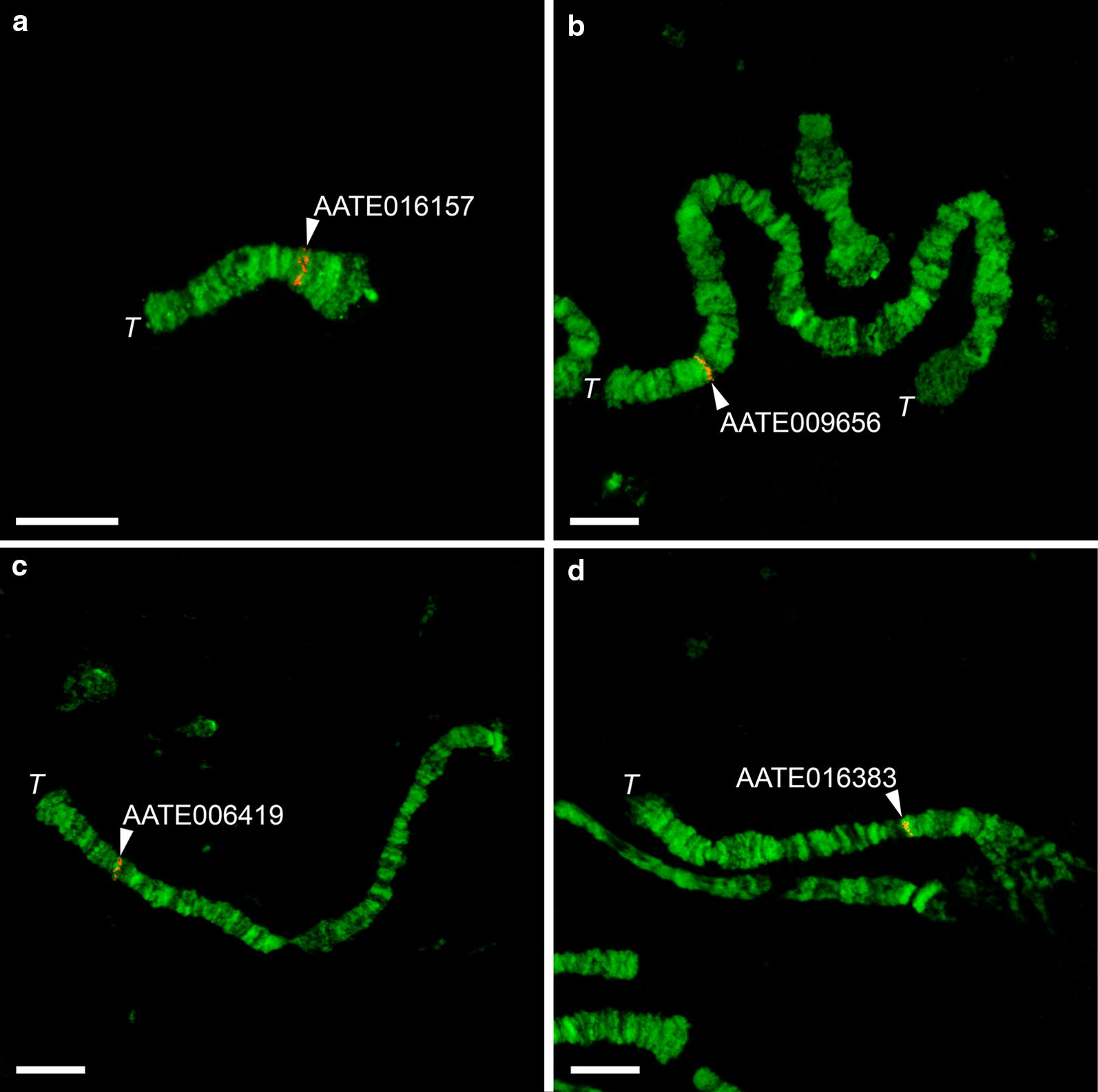
Examples of FISH with ovarian nurse cells polytene chromosomes of *Anopheles sacharovi*. Positions of four genes AATE009010 (**a**), AATE009656 (**b**), AATE006419 (**c**), and AATE016383 (**d**) are shown by arrows on the X chromosome and the 2R, 3R, and 3L arms, respectively. The scale bar represents 50 μm. Telomeric regions are indicated as *T*

To test the utility of the new chromosome map developed for *An. sacharovi,* four probes designed based on *An. atroparvus* genes were hybridized to the ovarian nurse cell chromosomes of *An. sacharovi* using FISH. The genes AATE009010, AATE009656, AATE006419, and AATE016383 were selected from the scaffolds previously mapped to the X chromosome, 2R arm, 3R arm, and 3L arm of *An. atroparvus*, respectively [32, 33]. Each DNA probe hybridized to the chromosomes of *An. sacharovi* (Fig. 3), and their positions were placed on the chromosome map (Fig. 2). Gene AATE009010 was mapped to region 1B in the chromosome X, gene AATE009656 was assigned to region 7B in the 2R arm, gene AATE006419 was anchored to region 23C/24A in the 3R arm, and gene AATE016383 was placed in the region 35B in the 3L arm. Thus, all markers were successfully mapped to a specific position in the newly developed map of *An. sacharovi* chromosomes, confirming the utility of this map for physical mapping.

### A cytotaxonomic approach to identification of *Anopheles sacharovi*

**Fig. 4:**
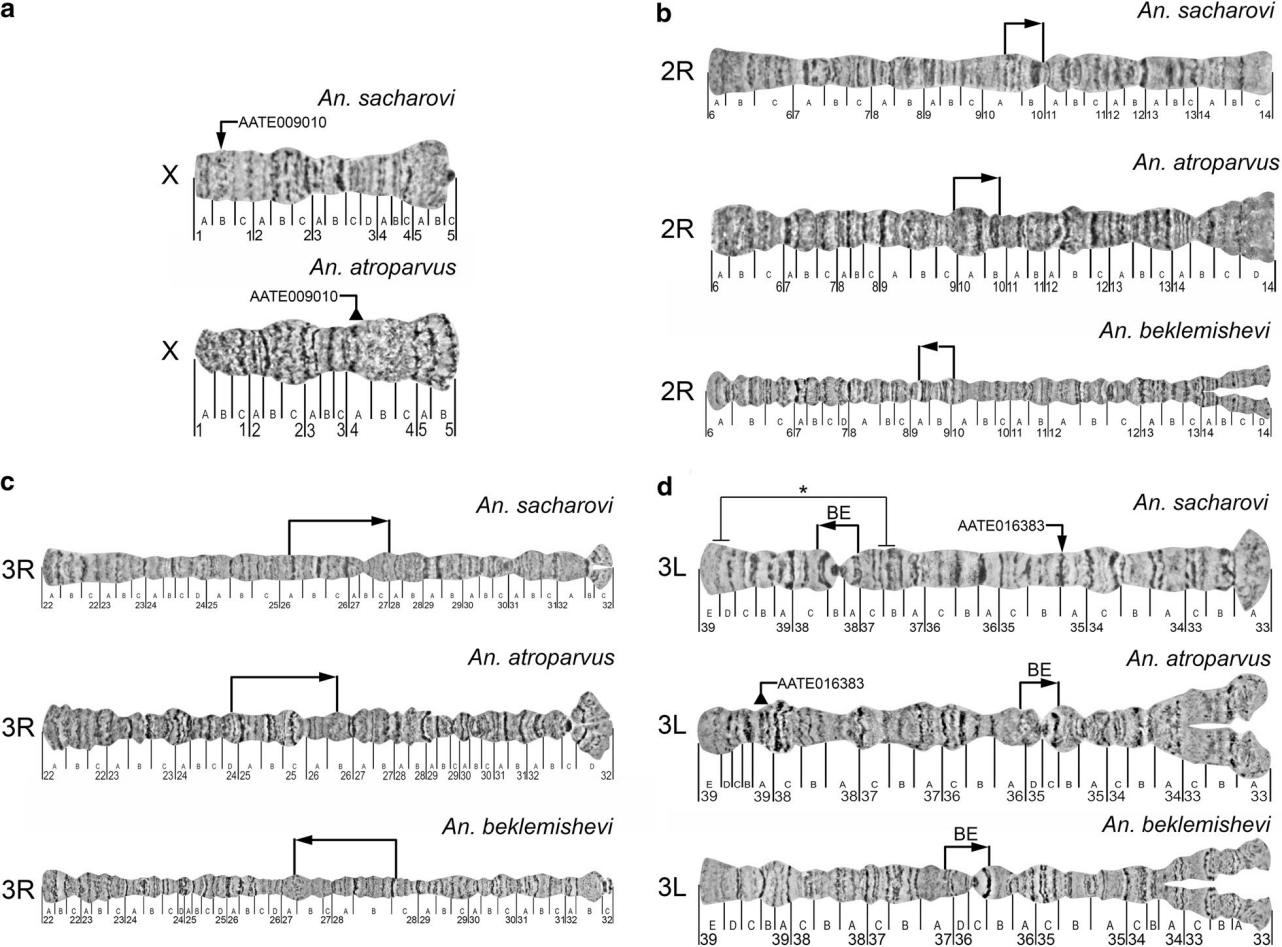
Cytotaxonomic features in nurse cell polytene chromosomes of *Anopheles sacharovi*, *Anopheles atroparvus* and *Anopheles beklemishevi*. Positions of the DNA probes AATE009010 (**a**) and AATE016383 (**d**) on chromosome X and 3L arm are shown by arrows. Chromosomal landmarks on chromosomes 2R (**b**), 3R (**c**), and 3L (**d**) are indicated by brackets. Position of the inversion that differentiates *An. martinius* and *An. sacharovi* [15] is indicated by a bracket with an asterisk. BE stands for a ‘bird eye’ landmark

Polytene chromosomes can serve as cytotaxonomic tools for species identification [21]. The cytogenetic map for *An. sacharovi* is the third map among the recently developed maps for members of the Maculipennis group. The other two maps were developed for *An. atroparvus* [33] and *An. beklemishevi* [43]. The comparison of polytene chromosomes among the species allowed to identify species-specific features that can be used for cytotaxonomic diagnostics. The overall quality of the banding pattern in the polytene chromosomes of the ovarian nurse cells was species-specific (Fig. 4). For example, chromosomes of *An. sacharovi* and *An. atroparvus* are characterized by fuzzy boundaries between bands and interbands. In contrast, chromosomes of *An. beklemishevi* have a clear banding pattern with sharp band-interband boundaries that are similar to the structure of the salivary gland chromosomes. Although all 3 species have similar landmarks in the middle of the arms, the positions and orientations of these landmarks in the chromosomes vary among species because of the presence of fixed pericentric inversions. For interspecies comparison, traditional nomenclature was followed in which the banding pattern of *An. atroparvus* is considered as standard for the entire species group [21]. The most robust landmark for the species diagnostics is a ‘bird eye’ landmark in the 3L arm. This landmark is organized as a tandem of two dark bands: a dot-like band and a dark crescent-shaped band (Fig. 4d). In *An. atroparvus*, the ‘bird eye’ is located close to the centromere in a standard orientation: the dot-like band is followed by the crescent-shaped band. By contrast, in *An. sacharovi,* the ‘bird eye’ is located closer to the telomere and has an inverted orientation. In *An. beklemishevi,* this landmark moved almost to the middle of the arm and has a standard orientation. Figure 4d shows the positions of the inversion that differentiates *An. sacharovi* and *An. martinius.* In the latter species, the ‘bird eye’ is located even closer to the telomere and has a standard orientation. The landmarks on chromosomes 2R (Fig. 4b) and 3R (Fig. 4c) have reverse orientations in *An. beklemishevi* in comparison with both *An. atroparvus* and *An. sacharovi*. The re-arrangements in the X chromosome (Fig. 4a) and 3L arm (Fig. 4d) between the species are seen based on the position of 2 genes, AATE009010 and AATE016383, that are located on opposite sides of the chromosomes in *An. sacharovi* and *An. atroparvus.*

**Fig. 5:**
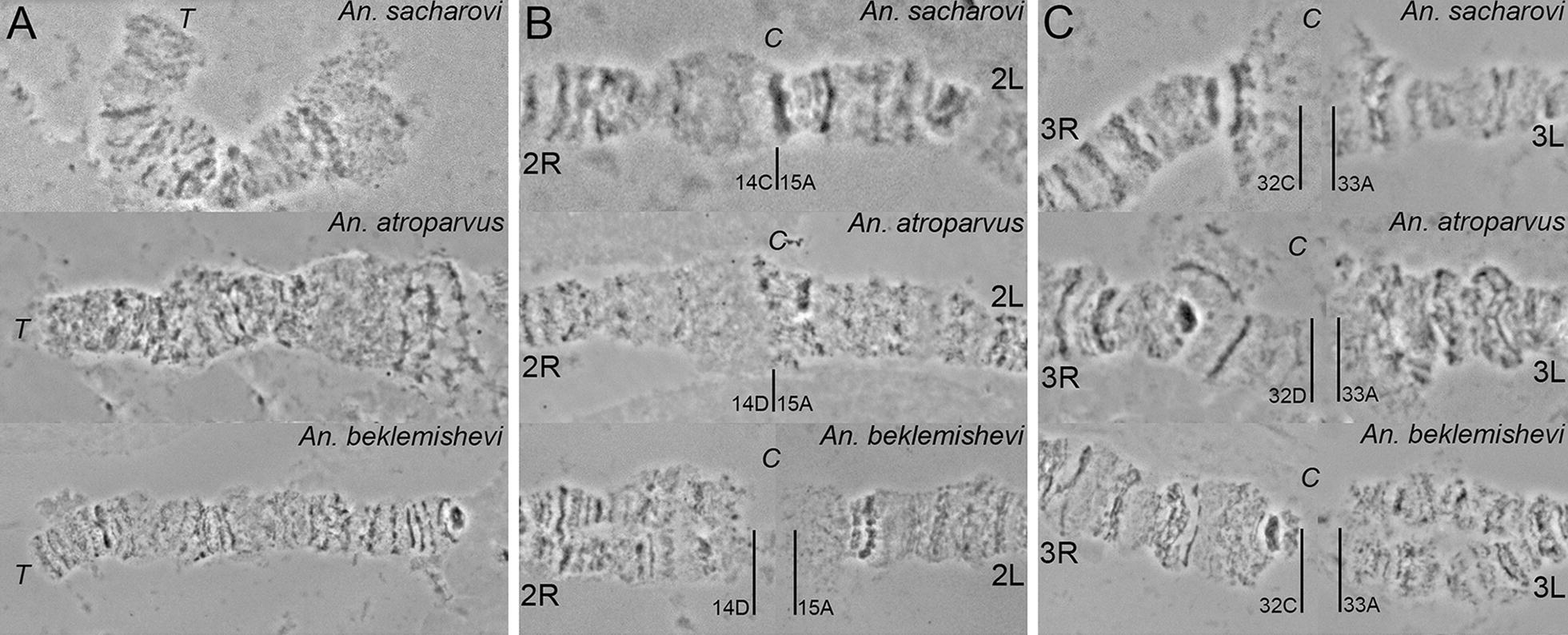
Cytotaxonomic features in pericentric regions of nurse cells polytene chromosomes in *Anopheles sacharovi*, *Anopheles atroparvus* and *Anopheles beklemishevi*. *T* stands for telomeric regions in chromosome X (**A**) and *C* stands for pericentromeric regions in chromosomes 2 (**B**) and 3 (**C**). Solid lines indicate boundaries between chromosome arms in the pericentromeric regions. The distances between chromosome arms are set arbitrary

In addition to fixed chromosomal inversions between the species, pericentomeric regions of the chromosomes have distinct species-specific features in *An. sacharovi*, *An. atroparvus* and *An. beklemishevi* [41]. For example, both *An. sacharovi* and *An. beklemishevi* have a compact dot-like pericentromeric band in the X chromosome, whereas in *An. atroparvus,* the X chromosome has three thin line-shape bands (Fig. 5A). On the other hand, the pericentric regions of the X chromosome in *An. atroparvu*s and *An. sacharovi* consist of a diffuse chromatin, whereas this part of the X chromosome in *An. beklemishevi* has a regular banding pattern. The homologous chromosomes 2 in *An. sacharovi* and *An. atroparvus* are paired in pericentromeric regions. The pericentric region in 2R and 2L arms in *An. beklemishevi* is asynaptic, and the arms of chromosome 2 have a fragile connection because of their attachment to the nuclear envelope [42] that is usually disrupted by squashing during chromosome preparation (Fig. 5B). Similarly, fragile pericentric regions are characteristics of chromosome 3 in all 3 species. However, in *An. beklemishevi*, this region does not form a fan-like structure as is seen in *An. sacharovi* and *An. atroparvus* (Fig. 5C). Thus, this study demonstrated that cytogenetic maps could serve for the cytotaxonomic diagnostic of *An. sacharovi* and other species from the Maculipennis group.

## Discussion

This study developed a high-resolution map for unstained polytene chromosomes from the ovarian nurse cells of *An. sacharovi*. Chromosome images were developed using phase-contrast microscopy and high-resolution digital photography that provide a detailed banding pattern of the polytene chromosomes. The first chromosome map of *An. sacharovi* was developed for the polytene chromosomes from the chromosomes of salivary glands [15]. Those chromosomes were stained with lacto-aceto-orcein and imaged using film photography. That technique provided too contrasted banding patterns of the chromosomes because of the overstaining of the large bands under staining of thin bands. Also, using ovarian nurse cell chromosomes in this study helped to avoid the loss of detail in the structure of the pericentromeric regions, usually affected by under-replication in salivary glands due to their heterochromatic nature [44].

The cytogenetic map that was developed for *An. sacharovi* is one of 12 chromosome maps constructed for anopheline species using digital imaging technology, since 2000 (Table 2). A special emphasis has been placed on cytogenetic map construction for the dominant vectors of malaria from different parts of the world [3, 45]. Cytogenetic maps are now available for the malaria vectors in Africa: *An. gambiae* [36], *Anopheles funestus* [46] and *Anopheles nili* [35]; in Asia: *An. stephensi* [47] and *Anopheles sinensis* [48]; and in America: *Anopheles albimanus* [49] and *Anopheles darlingi* [50, 51]. Chromosome maps have been used to identify and describe inversion polymorphism in natural populations of *An. funestus* [46], *An. nili* [35] and *An. darlingi* [50, 51]. Physical maps of microsatellite markers have been used in population genetic studies of *An. funestus* [52], *An. stephensi* [53] and *An. nili* [54]. Although inversion polymorphism has not been reported for *An. sacharovi,* the availability of the cytogenetic map may help to discover polymorphic inversions in this species in the future. Considering the limited number of population studies conducted for *An. sacharovi*, as well as the large geographical range of this mosquito, spreading from southern Europe to the Middle East, such discovery is quite possible.

**Table 2:** The development of chromosome maps for malaria mosquitoes

Mosquito species	Chromosome source	Cytogenetic map year/reference	Physical map year/reference	Genome map year/reference
*An. albimanus*	Salivary glands	2000 [49], 2017 [32]	2000 [49]	2017 [32]
*An. atroparvus*	Ovarian nurse cells	2015 [33]	2009 [57]	2015 [33], 2018 [55]
*An. beklemishevi*	Ovarian nurse cells	2018 [43]		
*An. darlingi*	Salivary glands	2010 [50], 2016 [51]		
*An. funestus*	Ovarian nurse cells	2001 [46]	2004 [52]	2002 [58]
*An. gambiae*	Ovarian nurse cells	2010 [36]		2002 [27], 2007 [31], 2010 [36]
*An. lesteri*	Salivary glands	2016 [59]	2016 [59]	
*An. nili*	Ovarian nurse cells	2011 [35]	2011 [35], 2012 [54]	
*An. ovengensis*	Ovarian nurse cells	2013 [60]		
*An. sacharovi*	Ovarian nurse cells	Current study		
*An. sinensis*	Salivary glands	2014 [48]	2014 [48]	2017 [56]
*An. stephensi*	Ovarian nurse cells	2006 [47]	2010 [39], 2010 [34], 2011 [53]	2014 [29]

Another reason that stimulated the recent interest in cytogenetic research in mosquitoes is the need to develop chromosome-based physical genome maps to enhance the quality of genome assemblies (Table 2). A superior physical genome map has been developed based on polytene chromosomes for the African malaria vector *An. gambiae* [36]. The genome of this species has been mapped by in situ hybridization of ~ 2000 bacterial artificial chromosome (BAC) clones [27]. The map has been improved by additional mapping of cDNA probes that allowed a better coverage of the heterochromatic regions [31]. Finally, the genome coordinates have been placed on the cytogenetic photomap developed using a high-pressure technique for squashing chromosomal preparation [36]. This map assigns 84.3% of the AgamP3 assembly to the chromosomes with coordinates spaced at 0.5–1 Mb intervals. A remarkably high-coverage genome map has been developed for the Neotropical vector of malaria *An. albimanus* [32] and for the European vector *An. atroparvus* [55]. These maps include 98.2 and 89.6% of their genome assemblies, respectively, and represent the most complete genome maps developed for mosquitoes to date. Lower coverage physical maps have been developed for dominant Asian malaria vectors, *An. stephensi* [29, 39] and *An. sinensis* [56], and for a major malaria vector in Africa, *An. funestus* [28]. In this study, physical mapping was performed for 4 orthologous genes on *An. sacharovi* chromosomes, for which location in the *An. atroparvus* genome and chromosomes is known. This study demonstrates that the *An. sacharovi* map is suitable for physical mapping and can serve as a tool for the development of a high-quality genome assembly for this species.

In addition, chromosome banding patterns of the *An. sacharovi* map were compared with other chromosome maps that were recently developed for the members of the Maculipennis group, *An. atroparvus* [33] and *An. beklemishevi* [43]. The comparison clearly demonstrated that the chromosome map of *An. sacharovi* can be utilized as a tool for the cytotaxonomic diagnostic of these 3 species and for a sister taxon *An. martinius* [15]. This comparison of the chromosomal banding patterns and positions of the physically mapped DNA probes also indicated that chromosome-banding patterns are not always reliable in determining chromosomal re-arrangements between the species. For example, FISH data demonstrated that the gene AATE009010 of *An. atroparvus* and its orthologue in *An. sacharovi* localize in two different positions in the X chromosome; the difference is likely caused by paracentric inversions that are not evident cytogenetically (Fig. 4a). The position of AATE016383 in 3L arm was also different in *An. sacharovi* and *An. atroparvus* (Fig. 4d), suggesting a more complex pattern of chromosomal re-arrangements in the 3L arm between the species from the Maculipennis group than was previously thought based on chromosomal banding patterns [21]. Thus, additional physical mapping is required to better understand chromosomal evolution in the Maculipennis group of malaria mosquitoes.

## Conclusions

This study reports the development of a standard photomap for the ovarian nurse cell chromosomes of *An. sacharovi*, one of the most dangerous malaria vectors in Europe and Middle East. The suitability of this map for physical mapping is demonstrated by successful positioning of 4 DNA probes on the map using FISH. In addition, cytotaxonomic features for identification of *An. sacharovi* and 2 other species from the Maculipennis group: *An. atroparvus* and *An. beklemishevi*, were identified and described. The cytogenetic map constructed in this study will help to create a chromosome-based genome assembly for this mosquito and will further stimulate the development of genomics-based strategies for vector control.

## Abbreviations

DNA: deoxyribonucleic acid
dATP: deoxyadenosine triphosphate
dGTP: deoxyguanosine triphosphate
dCTP: deoxycytidine triphosphate
dNTP: deoxy nucleoside triphosphate
FISH: fluorescence in situ hybridization
ITS2: internal transcribe spacer 2
PCR: polymerase chain reaction

## References

[1] Favr VV (1903). An attempt to study malaria from the viewpoint of sanitation.

[2] Harbach RE (2004). The classification of genus *Anopheles* (Diptera: Culicidae): a working hypothesis of phylogenetic relationships. Bull Entomol Res.

[3] Sinka ME, Bangs MJ, Manguin S, Coetzee M, Mbogo CM, Hemingway J (2010). The dominant *Anopheles* vectors of human malaria in Africa, Europe and the Middle East: occurrence data, distribution maps and bionomic precis. Parasit Vectors.

[4] Hanafi-Bojd AA, Azari-Hamidian S, Vatandoost H, Charrahy Z (2011). Spatio-temporal distribution of malaria vectors (Diptera: Culicidae) across different climatic zones of Iran. Asian Pac J Trop Med.

[5] Vatandoost H, Ashraf H, Lak SH, Mahdi RE, Abai MR, Nazari M (2003). Factors involved in the re-emergence of malaria in borderline of Iran, Armenia, Azerbaijan and Turkey. Southeast Asian J Trop Med Public Health.

[6] Oshaghi MA, Vatandoost H, Gorouhi A, Abai MR, Madjidpour A, Arshi S (2011). Anopheline species composition in borderline of Iran-Azerbaijan. Acta Trop.

[7] Hantosh HA, Hassan HM, Ahma B, Al-fatlawy A (2012). Mosquito species geographical distribution in Iraq 2009. J Vector Borne Dis.

[8] Ozbilgina A, Topluoglu S, Es S, Islek E, Mollahaliloglu S, Erkoc Y (2011). Malaria in Turkey: successful control and strategies for achieving elimination. Acta Trop.

[9] Bezzhonova OV, Babuadze GA, Gordeev MI, Goriacheva, II, Zvantsov AB, Ezhov MN, et al. Malaria mosquitoes of the *Anopheles maculipennis* (Diptera, Culicidae) complex in Georgia (in Russian). Med Parazit (Mosk). 2008:32–6. 18819426

[10] Keshish’ian A, Gordeev MI, Bezzhonova OV, Goriacheva, II, Zvantsov AB, Davidiants VA, et al. Genetic analysis of malaria mosquitoes of *Anopheles maculipennis* (Diptera, Culicidae) complex from Armenia (in Russian). Med Parazit (Mosk). 2009:24–8. 19827512

[11] Keshishian A, Aleksanian Iu T, Manukian DV. The environmental and epidemiological aspects of malaria in Armenia and prospects for its eradication (in Russian). Med Parazit (Mosk). 2007:37–9. 17657955

[12] Gordeev MI, Bezzhonova OV, Goriacheva, II, Shaikevich EV, Zvantsov AB, Mamedov S, et al. Molecular genetic analysis of malaria mosquitoes of the *Anopheles maculipennis* (Diptera, Culicidae) complex in Azerbaijan (in Russian). Med Parazit (Mosk). 2010:43–5. 21395043

[13] Vatandoost H, Abai MR (2012). Irritability of malaria vector, *Anopheles sacharovi* to different insecticides in a malaria-prone area. Asian Pac J Trop Med.

[14] Salahi-Moghaddam A, Khoshdel A, Dalaei H, Pakdad K, Nutifafa GG, Sedaghat MM (2017). Spatial changes in the distribution of malaria vectors during the past 5 decades in Iran. Acta Trop.

[15] Stegniy VN (1976). Identification of chromosomal forms in malaria mosquito *Anopheles sacharovi* (in Russian). Tsitol.

[16] Shingarev NI (1926). New information on Culicidae of the USSR (in Russian). Russian J Trop Med.

[17] Zhelokhovtsev AN (1937). Notes in systematics of genus *Anopheles* (in Russian). Med Parazit and Parazit Bolezni.

[18] Stegniy VN (1980). Reproductive relationships in malaria mosquitoes Anopheles from Maculipennis compex (in Russian). Zool Zhur.

[19] White GB (1978). Systematic reappraisal of the *Anopheles maculipennis* complex. Mosq Systemat.

[20] Marinucci M, Romi R, Mancini P, Di Luca M, Severini C (1999). Phylogenetic relationships of seven palearctic members of the maculipennis complex inferred from ITS2 sequence analysis. Insect Mol Biol.

[21] Stegniy VN (1981). Genetic basis of evolution in malaria mosquitoes *Anopheles* from Maculipennis complex (Diptera, Culicidae). 1. Chromosome-based phylogenetic relationships (in Russian). Zool Zhur.

[22] Kampen H (2005). The ITS2 ribosomal DNA of *Anopheles beklemishevi* and further remarks on the phylogenetic relationships within the *Anopheles maculipennis* group of species (Diptera: Culicidae). Parasitol Res.

[23] Djadid ND, Gholizadeh S, Tafsiri E, Romi R, Gordeev M, Zakeri S (2007). Molecular identification of Palearctic members of *Anopheles maculipennis* in northern Iran. Malar J.

[24] Carvalho DO, McKemey AR, Garziera L, Lacroix R, Donnelly CA, Alphey L (2015). Suppression of a field population of *Aedes aegypti* in Brazil by sustained release of transgenic male mosquitoes. PLoS Negl Trop Dis.

[25] Yamamoto DS, Sumitani M, Kasashima K, Sezutsu H, Matsuoka H (2016). Inhibition of malaria infection in transgenic Anopheline mosquitoes lacking salivary gland cells. PLoS Pathog.

[26] Gantz VM, Jasinskiene N, Tatarenkova O, Fazekas A, Macias VM, Bier E (2015). Highly efficient Cas9-mediated gene drive for population modification of the malaria vector mosquito *Anopheles stephensi*. Proc Natl Acad Sci US.

[27] Holt RA, Subramanian GM, Halpern A, Sutton GG, Charlab R, Nusskern DR (2002). The genome sequence of the malaria mosquito *Anopheles gambiae*. Science.

[28] Neafsey DE, Waterhouse RM, Abai MR, Aganezov SS, Alekseyev MA, Allen JE (2015). Mosquito genomics. Highly evolvable malaria vectors: the genomes of 16 Anopheles mosquitoes. Science.

[29] Jiang X, Peery A, Hall AB, Sharma A, Chen XG, Waterhouse RM (2014). Genome analysis of a major urban malaria vector mosquito, *Anopheles stephensi*. Genome Biol.

[30] Lawniczak MK, Emrich SJ, Holloway AK, Regier AP, Olson M, White B (2010). Widespread divergence between incipient *Anopheles gambiae* species revealed by whole genome sequences. Science.

[31] Sharakhova MV, Hammond MP, Lobo NF, Krzywinski J, Unger MF, Hillenmeyer ME (2007). Update of the *Anopheles gambiae* PEST genome assembly. Genome Biol.

[32] Artemov GN, Peery AN, Jiang X, Tu Z, Stegniy VN, Sharakhova MV (2017). The physical genome mapping of *Anopheles albimanus* corrected scaffold misassemblies and identified interarm rearrangements in genus Anopheles. G3 (Bethesda).

[33] Artemov GN, Sharakhova MV, Naumenko AN, Karagodin DA, Baricheva EM, Stegniy VN (2015). A standard photomap of ovarian nurse cell chromosomes in the European malaria vector *Anopheles atroparvus*. Med Vet Entomol.

[34] Xia A, Sharakhova MV, Leman SC, Tu Z, Bailey JA, Smith CD (2010). Genome landscape and evolutionary plasticity of chromosomes in malaria mosquitoes. PLoS ONE.

[35] Sharakhova MV, Antonio-Nkondjio C, Xia A, Ndo C, Awono-Ambene P, Simard F (2011). Cytogenetic map for *Anopheles nili*: application for population genetics and comparative physical mapping. Infect Genet Evol.

[36] George P, Sharakhova MV, Sharakhov IV (2010). High-resolution cytogenetic map for the African malaria vector *Anopheles gambiae*. Insect Mol Biol.

[37] National Center for Biotechnology Information. 2018. http://www.ncbi.nlm.nih.gov. Accessed 16 Jul 2018.

[38] VectorBase. 2018. https://www.vectorbase.org. Accessed 28 Jun 2018.

[39] Sharakhova MV, Xia A, Tu Z, Shouche YS, Unger MF, Sharakhov IV (2010). A physical map for an Asian malaria mosquito, *Anopheles stephensi*. Am J Trop Med Hyg.

[40] Sharakhova MV, George P, Timoshevskiy V, Sharma A, Peery A, Sharakhov I, Group CPTF (2015). Mosquito (Diptera). Protocols for cytogenetic mapping of arthropod genomes.

[41] Sharakhova MV, Stegniy VN (1997). Interspecies differences in the ovarian trophocyte precentromere heterochromatin structure and evolution of the malaria mosquito complex *Anopheles maculipennis* (in Russian). Genetika.

[42] Stegniy VN (1979). Reorganization of the structure of interphase nuclei during the onto- and phylogenesis of malaria mosquitoes (in Russian). Dok Acad Nauk SSSR.

[43] Artemov GN, Gordeev MI, Kokhanenko AA, Moskaev AV, Velichevskaya AI, Stegniy VN (2018). A standard photomap of ovarian nurse cell chromosomes and inversion polymorphism in *Anopheles beklemishevi*. Parasit Vectors.

[44] Zhimulev IF (1998). Polytene chromosomes, heterochromatin, and position effect variegation. Adv Genet.

[45] Sinka ME, Rubio-Palis Y, Manguin S, Patil AP, Temperley WH, Gething PW (2010). The dominant *Anopheles* vectors of human malaria in the Americas: occurrence data, distribution maps and bionomic precis. Parasit Vectors.

[46] Sharakhov IV, Sharakhova MV, Mbogo CM, Koekemoer LL, Yan G (2001). Linear and spatial organization of polytene chromosomes of the African malaria mosquito *Anopheles funestus*. Genetics.

[47] Sharakhova MV, Xia A, McAlister SI, Sharakhov IV (2006). A standard cytogenetic photomap for the mosquito *Anopheles stephensi* (Diptera: Culicidae): application for physical mapping. J Med Entomol.

[48] Liang J, Sharakhova MV, Lan Q, Zhu H, Sharakhov IV, Xia A (2014). A standard cytogenetic map for *Anopheles sinensis* and chromosome arm homology between the subgenera Anopheles and Cellia. Med Vet Entomol.

[49] Cornel AJ, Collins FH (2000). Maintenance of chromosome arm integrity between two Anopheles mosquito subgenera. J Hered.

[50] Rafael MS, Rohde C, Bridi LC, Valente Gaiesky VL, Tadei WP (2010). Salivary polytene chromosome map of *Anopheles darlingi*, the main vector of neotropical malaria. Am J Trop Med Hyg.

[51] Cornel AJ, Brisco KK, Tadei WP, Secundino NF, Rafael MS, Galardo AK (2016). *Anopheles darlingi* polytene chromosomes: revised maps including newly described inversions and evidence for population structure in Manaus. Mem Inst Oswaldo Cruz.

[52] Sharakhov I, Braginets O, Grushko O, Cohuet A, Guelbeogo WM, Boccolini D (2004). A microsatellite map of the African human malaria vector *Anopheles funestus*. J Hered.

[53] Kamali M, Sharakhova MV, Baricheva E, Karagodin D, Tu Z, Sharakhov IV (2011). An integrated chromosome map of microsatellite markers and inversion breakpoints for an Asian malaria mosquito, *Anopheles stephensi*. J Hered.

[54] Peery A, Sharakhova MV, Antonio-Nkondjio C, Ndo C, Weill M, Simard F (2011). Improving the population genetics toolbox for the study of the African malaria vector *Anopheles nili*: microsatellite mapping to chromosomes. Parasit Vectors.

[55] Artemov GN, Bondarenko SM, Naumenko AN, Stegniy VN, Sharakhova MV, Sharakhov IV (2018). Partial-arm translocations in evolution of malaria mosquitoes revealed by high-coverage physical mapping of the *Anopheles atroparvus* genome. BMC Genomics.

[56] Wei Y, Cheng B, Zhu G, Shen D, Liang J, Wang C (2017). Comparative physical genome mapping of malaria vectors *Anopheles sinensis* and *Anopheles gambiae*. Malar J.

[57] Grushko OG, Sharakhova MV, Stegnii VN, Sharakhov IV (2009). Molecular organization of heterochromatin in malaria mosquitoes of the *Anopheles maculipennis* subgroup. Gene.

[58] Sharakhov IV, Serazin AC, Grushko OG, Dana A, Lobo N, Hillenmeyer ME (2002). Inversions and gene order shuffling in *Anopheles gambiae* and *A. funestus*. Science.

[59] Liang J, Cheng B, Zhu G, Wei Y, Tang J, Cao J (2016). Structural divergence of chromosomes between malaria vectors *Anopheles lesteri* and *Anopheles sinensis*. Parasit Vectors.

[60] Sharakhova MV, Peery A, Antonio-Nkondjio C, Xia A, Ndo C, Awono-Ambene P (2013). Cytogenetic analysis of *Anopheles ovengensis* revealed high structural divergence of chromosomes in the *Anopheles nili* group. Infect Genet Evol.

